# The Genetic Structure of *Staphylococcus aureus* Populations from the Southwest Pacific

**DOI:** 10.1371/journal.pone.0100300

**Published:** 2014-07-08

**Authors:** Stephen R. Ritchie, Mark G. Thomas, Paul B. Rainey

**Affiliations:** 1 NZ Institute for Advanced Study and Allan Wilson Centre for Molecular Ecology and Evolution, Massey University, Auckland, New Zealand; 2 Department of Molecular Medicine and Pathology, University of Auckland, Auckland, New Zealand; 3 Max Planck Institute for Evolutionary Biology, Plön, Germany; University of California, San Francisco, United States of America

## Abstract

The genetic structure of *Staphylococcus aureus* populations sampled from diverse regions of the globe have been the subject of numerous investigations. Here we describe the structure of *S. aureus* populations collected from the Southwest Pacific. Multi-locus sequence typing was performed on 467 isolates obtained from people with nasal colonization or bacteremia in Auckland (NZ), and patients predominantly affected by skin and soft tissue infection in Samoa, Fiji and Tonga. The predominant sequence types (STs) varied between Auckland (ST5), Fiji (ST30), and Samoa (ST1), however, the overall genetic diversity within each region did not differ significantly between locations. Divergent Clonal Complex 75 (CC75) strains were isolated in Auckland and Fiji. When diversity of the Southwest Pacific populations was compared with those previously described from Asia, Europe, North America and Africa no significant differences were detected. With the exception of CC75 strains, the global collection of *S. aureus* encompasses relatively little diversity, with novel STs arising locally from a small number of widespread lineages.

## Introduction

*Staphylococcus aureus* is a ubiquitous human commensal and pathogen that causes disease worldwide. *S. aureus* possesses a broad collection of virulence factors that aid in colonization, tissue invasion and immune evasion. Many of these factors have high specificity for humans [1]. As a result, the global epidemiology of *S. aureus* primarily reflects the *S. aureus* strains that infect humans. Although infection of human hosts has been a focus of attention, there is growing recognition that *S. aureus* is also associated with domesticated and wild animals. Studies of domesticated animals suggest a host switch from humans occurred in the past [2], [3]; however, there is evidence for the existence of divergent *S. aureus* strains associated with non-domesticated animals, including non-human primates [4], [5].

The development of multi-locus sequence typing (MLST) has facilitated analysis of the genetic structure of numerous pathogens, including *S. aureus.* The first detailed study examined *S. aureus* isolates obtained from patients with disease and from people with asymptomatic nasal colonization in Oxford, England [6]. This study showed a population structure comprised of a limited number of related clonal complexes (CCs), within which new sequence types (STs) arise, primarily by point mutation. Although a link between horizontal gene transfer and virulence has been noted [7], recombination appears to have played a relatively minor role in shaping population structure [6].

Since the England study, the MLST-derived genetic structure of *S. aureus* populations has been examined in Europe, North America, Asia and Africa [6], [8], [9], [10], [11], [12], [13], [14]. Each study has brought to light novel STs (STs that have not been described previously); however, for the most part predominant CCs are similar within broad geographic regions. For example, CC30 isolates are most common across Europe [6], [9], while CC121 isolates are most frequently encountered in Asia [8], [9]. Despite these regional differences, STs from common CCs are globally dispersed.

While the predominant *S. aureus* STs isolated in all countries are relatively closely related, highly divergent *S. aureus* STs belonging to CC75 have been isolated from residents of Cambodia, Northern Australia and a remote village in French Guiana [9], [11], [15], [16]. The genetic distance between CC75 *S. aureus* STs and conventional *S. aureus* STs led Holt, et al [17], to suggest that CC75 STs represent a distinct species (*S. argenteus*). However, CC75 STs are phylogenetically more similar to conventional *S. aureus* than to other staphylococcal species (e.g.CC75 STs and conventional *S. aureus* STs share identical 16srRNA sequences).

In New Zealand, the incidence of *S. aureus* disease is higher among Māori and Pacific people than among people of European or other ethnicities [18], [19], [20], [21]; and available evidence suggests the incidence of disease is high in indigenous people in Pacific nations [22]. Furthermore the incidence of disease caused by community-acquired MRSA is higher in Māori, Pacific people and Aboriginal Australians than in people of European or other ethnicities [20], [23], [24]. This led us to question whether the strains of *S. aureus* colonizing and/or causing disease in people in New Zealand and other Southwest Pacific nations – a region remote from previous studies – differ from the strains previously identified in Africa, Asia, Europe and North America.

Here, we report the population structure of *S. aureus* isolated from humans in the Southwest Pacific. We compare the diversity of *S. aureus* populations from neighboring Southwest Pacific Island nations, and contrast this with the genetic diversity described in studies conducted around the globe.

## Methods

### Participants and *S. aureus* strains

Ethical approval for the collection of isolates in New Zealand was provided by the NZ Ministry of Health, Northern Y ethics committee; and written informed consent was obtained from patients with bacteremia and healthy volunteers. In Auckland, during 2007, 150 *S. aureus* isolates were prospectively obtained from consecutive adults (over 15 years of age) admitted to Auckland Hospital or Middlemore Hospital with *S. aureus* bacteremia. The 150 isolates included 61 strains from patients with community-acquired infection, 32 strains from patients with healthcare-associated community-onset infection, and 57 strains from patients with nosocomial infection. During the same time period, 94 nasal carriage isolates were obtained from 457 healthy adult volunteers, recruited in shopping malls and other community venues, who reported no healthcare contact in the three months prior to recruitment.

Ethical approval for the collection of isolates in Samoa was provided by the Health Research council of Samoa and written informed consent was obtained from all participants [22]. During the summer of 2007–2008, 187 *S. aureus* isolates were obtained from 399 people with skin and soft tissue infections presenting to clinics in towns and villages throughout Samoa. 96 of these 187 isolates were randomly selected to represent the *S. aureus* population in Samoa.

Ethical approval for the collection of isolates in Fiji was provided by the Fijian Ministry of Health. The site of infection was the only clinical information obtained and informed consent was not required. During January to August 2008, 205 consecutive clinical isolates of *S. aureus* were obtained from patients presenting with a range of staphylococcal diseases to the Colonial War Memorial Hospital in Fiji's largest city, Suva. The majority of the strains (163/205, 80%) were isolated from patients with skin and soft tissue infections. 109 of these 205 isolates were randomly selected to represent the *S. aureus* population in Fiji.

In Tonga, during September 2007 to March 2008, 18 *S. aureus* isolates were obtained from patients presenting with a range of staphylococcal diseases to Vaiola Hospital, Nuku'a'lofa. The majority of the strains (12/18, 67%) were isolated from patients with skin and soft tissue infections. The site of infection was the only clinical information obtained and the Tongan Ministry of Health did not require formal ethical approval.

The identity of all isolates was confirmed by standard laboratory techniques. Two randomly selected colonies were separately inoculated into stock solution (35% v/v glycerol, 50 mM MgSO_4_, 125 µM TRIS pH8) and stored at −80°C.

### MLST and PCR primers

Genomic DNA for each isolate was extracted from one of the two stored colonies using the DNeasy kit (Qiagen, Hilden, Germany) according to the manufacturer's instructions. MLST was performed in accordance with established practice [25], [26]. The *aro* nucleotide PCR and sequencing failed on four occasions, and new primers *aro-fwd* 5′ - CCY ATT TCW CAT TCM TTR TCG C and *aro-rev* 5′ - CAT ACC MGC WGG TGT WGT RTT were used for PCR and sequencing with an annealing temperature of 54°C. Each amplified nucleotide sequence was trimmed to remove additional flanking nucleotides, and the remaining conventional MLST nucleotide sequences were used to assign allele designations and STs using the conventional MLST database (http://saureus.mlst.net/). CC designations containing double locus variants were assigned using eBURST in accordance with established practice [6]. Confirmation of novel STs (when a nucleotide sequence did not match a known allele in the database or when the combination of seven known alleles did not match a known ST) was provided by performing MLST on the second stored isolate.

### Intercontinental *S. aureus* population

The *S. aureus* population examined in the current study was compared with *S. aureus* populations described in previous human studies. These studies included isolates from asymptomatically colonised people in Chengdu, China [8] and Bamako, Mali [10]; asymptomatically colonised healthcare workers in Lausanne, Switzerland [27];and people with invasive staphylococcal disease or asymptomatic colonisation in Oxford, England [6], Florida, USA [28] and North Carolina, USA [12]. The allele designations and nucleotide sequences of the STs reported in these studies were obtained from the MLST database. Previously reported *S. aureus* populations were not included if they focused solely on MRSA (e.g. Ko, et al [29]), if MLST typing was only performed on selected isolates and the majority of strains were typed by another method (e.g. Uhlemann, et al [30];Breurec, et al [31]; Tong, et al [16]); or if ST designations were not reported in the publication and were not available from the MLST database (e.g. Ruimy, et al [9], [11]).

In summary, the intercontinental *S. aureus* population was represented by 334 strains (73 STs) isolated in England during 1997 and 1998; 147 strains (33 STs) isolated from children in China during 2005; 88 strains (20 STs) isolated in Mali during 2005; 379 strains (75 STs) isolated in North Carolina between 1994 and 2003; 93 strains (26 STs) isolated in Florida; 133 strains (37 STs) isolated in Switzerland; and 467 strains (85 STs) isolated from people in Southwest Pacific nations during 2007–2008. The concatenated MLST sequences of these strains are included in supplementary material (Data S1).

### Analysis

The nucleotide sequences of the seven housekeeping genes for each isolate were concatenated to form a single sequence of 3186 nucleotides. A comparison of the genetic diversity of the *S. aureus* population in each location was made using Simpson's index of diversity. Simpson's index estimates the probability that the ST of any two randomly drawn isolates are identical. goeBURST was used to group STs into CCs at the level of double-locus variants, using PHYLOVIZ v1.0 [32], [33].

The LDHAT likelihood permutation test was used to detect statistically significant recombination [34]. The Shimodaira-Hasegawa test [35] was used to compare the topologies of the individual gene trees with concatenated gene trees using TREE-PUZZLE v5.2 [36].

Bayesian phylogenies were reconstructed from individual and concatenated housekeeping gene nucleotide sequences using BEAST v.1.7.2 [37] and the substitution model described by Hasegawa, Kishino and Yano (HKY) [38]. We used a uniform rate of nucleotide heterogeneity (selected as the most likely evolutionary model using jMODELTEST 0.1.1 [39]) and a random local clock. Each Monte Carlo Markov Chain (MCMC) run consisted of 10^8^ iterations, with parameters sampled every 10^3^ iterations (the first 20% were discarded as burn-in). A maximum credibility consensus phylogeny was drawn from the combined results obtained from three separate runs.

Two dimensional multi-dimensional scaling maps were created from a genetic distance matrix using PRIMER 6.1.12 [40]. Each analysis was restarted 50 times to ensure that the best representation of the data, i.e. that with the lowest stress value, had been obtained. Statistical comparison of the genetic diversity of *S. aureus* populations from each country was performed using the permutational multivariate analysis of variance test (PERMANOVA), implemented in PERMANOVA 1.0.5 [41]. Comparisons of genetic diversity were made using a genetic distance matrix calculated from non-duplicate STs in each country's *S. aureus* population.

An intercontinental phylogenetic network was created from the BEAST derived majority rule consensus tree using Cytoscape 2.6.3 [42]. Due to marked differences in sample size between different studies, the network shows the proportion of isolates of each ST in each country rather than the total numbers of each ST.

## Results

### Predominant STs in the Southwest Pacific

**Figure 1:**
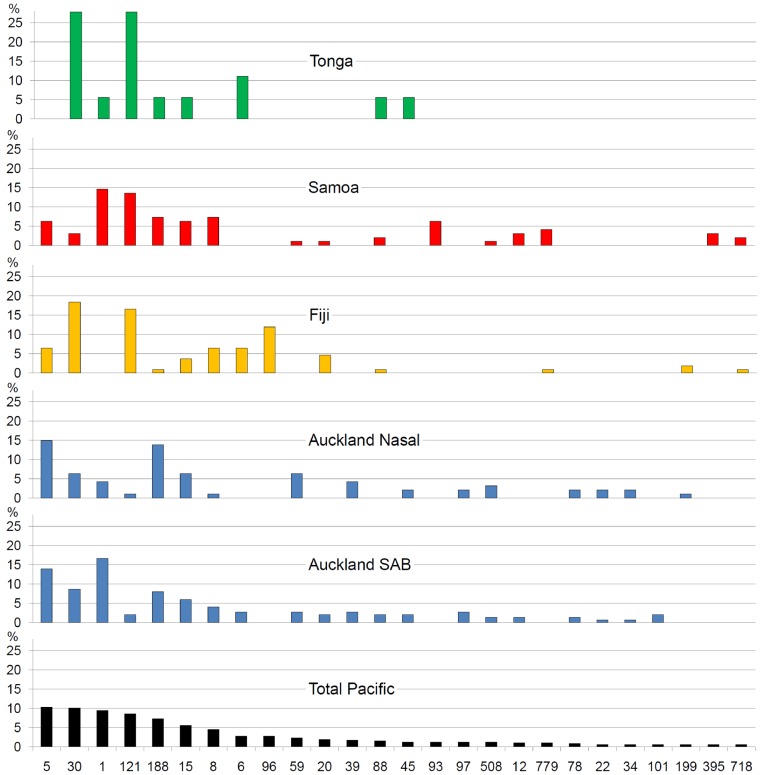
The frequency distribution (%) of the 26 most common *S. aureus* sequence types in Southwest Pacific Island nations. The figures show the distribution for the Total Southwest Pacific sample (n = 467), the Auckland SAB sample (n = 150), the Auckland nasal carriage sample (n = 94), the Fijian sample (n = 109), the Samoan sample (n = 96) and the Tongan sample (n = 18).

The 467 *S. aureus* isolates derived from four Southwest Pacific locations belonged to a total of 85 STs. Rarefaction analyses showed that each sample encompassed a high degree of diversity, especially at the level of clonal complexes (Figure S1). The ten most common STs contained the majority (297/467, 64%) of isolates (Figure 1). The predominant *S. aureus* STs varied between locations: notably ST1, the most common cause of bacteremia in Auckland and the most common cause of skin and soft tissue infection in Samoa, was not identified in the Fijian sample. ST30 was the most common ST in Fiji, but was the eighth most common ST in the Samoan population.

Six isolates belonging to CC75 were identified: four strains were isolated from people with asymptomatic nasal colonization in Auckland, and two from patients with skin and soft tissue infections in Fiji. None of these six isolates were methicillin resistant. These six isolates are closely related to CC75 strains isolated in Cambodia, Australia and French Guiana [9], [11], [15].

### Genetic diversity of *S. aureus* in the Southwest Pacific

**Figure 2:**
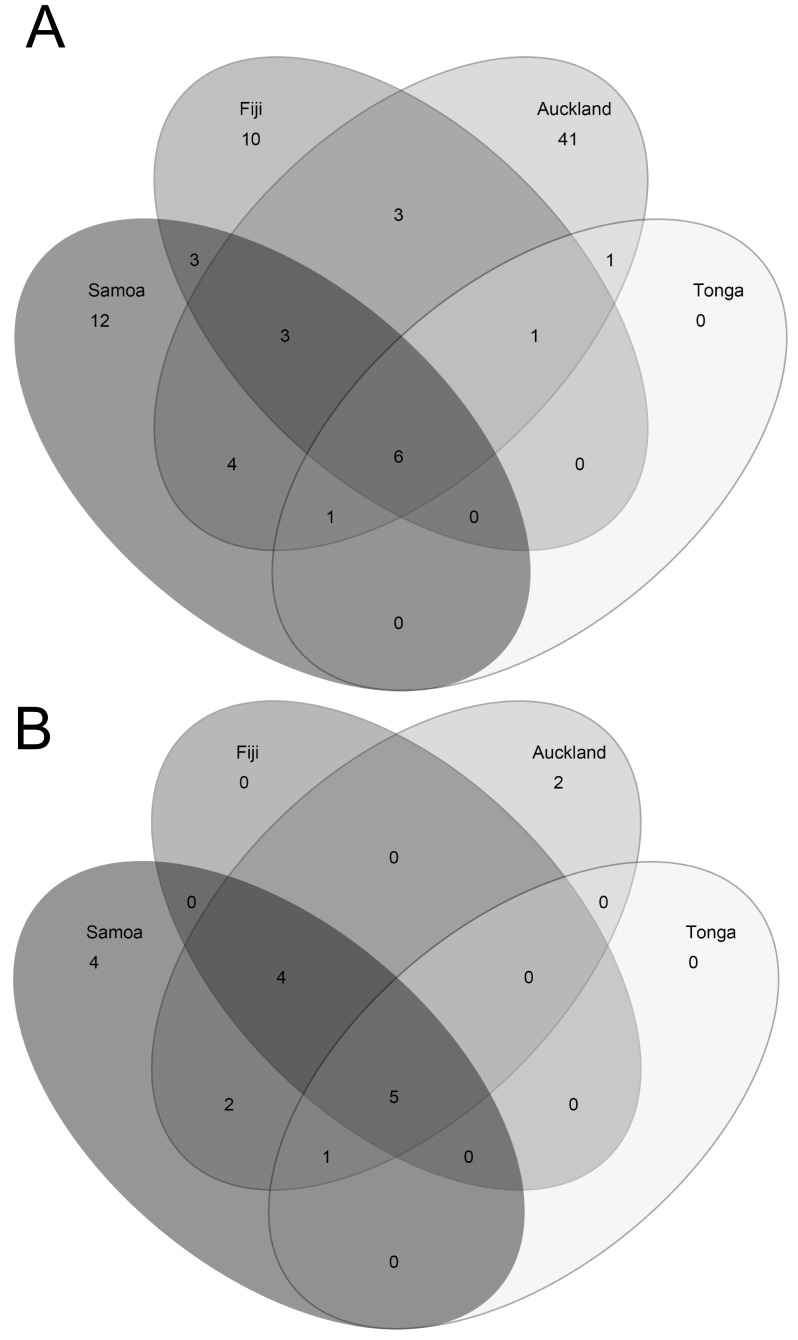
Venn diagrams showing (A) the degree of commonality of STs within Southwest Pacific locations; and (B) the degree of commonality of CCs within these locations.

Sixty STs were identified in the Auckland sample, 26 in the Fijian sample, 28 in the Samoan sample and 9 in the Tongan sample. The majority of these STs (63/85, 74%) were identified in only one location: 41 in Auckland, 10 in Fiji, and 12 in Samoa (Figure 2A). However, most of the STs identified in only one location were members of CCs that included other Southwest Pacific STs. Thus, more than half of the 29 CCs identified were found in more than one location (Figure 2B); and five CCs (CC1, 15, 30, 88 and 121) were identified in all four countries. Simpson's index of diversity did not differ between locations (range from Auckland 0.05, 95% CI 0.04–0.06 to Tonga 0.14, 95%CI 0.04–0.23).

### Recombination and the evolution of novel STs

Prior to reconstructing a phylogeny of Southwest Pacific-derived *S. aureus*, we sought evidence of recombination, because high levels of recombination can obscure phylogenetic information. There was no evidence of recombination within the nucleotide sequences of the individual seven MLST genes. However, the concatenated gene sequences for all seven MLST genes revealed that recombination events had occurred between the MLST genes (LDHAT test, *P*<0.001). In order to ensure that the recombination signal was not enhanced, or obscured, by inclusion of the divergent CC75 strains, analyses were performed in the presence and absence of these STs, which did not alter the results.

### Phylogeny of *S. aureus* in the Southwest Pacific

To determine whether recombination obscured the phylogenetic signal, individual gene trees were reconstructed for each locus and compared to the tree reconstructed from concatenated MLST gene sequences. None of the individual gene trees differed significantly from the concatenated tree (Shimodaira-Hasegawa test, *P*>0.05 for all pairwise tests).

Novel STs that did not match other STs contained within the MLST database comprised 61/467 (13%) of the STs in the Southwest Pacific sample. The proportion of novel STs was similar in each location (Auckland, 34/244 (14%); Fiji 17/109 (16%); Samoa 9/96 (9%); Tonga 1/18 (6%)). The Southwest Pacific phylogeny, rooted on the CC75 STs, demonstrates that these novel Southwest Pacific STs have arisen throughout the population (Figure S2). The phylogeny also reveals uniformly short terminal branch lengths for the majority of isolates.

### No difference between *S. aureus* populations from different countries within the Southwest Pacific

To test for evidence of biogeographic structure, multivariate analyses were performed by PERMANOVA analysis. This approach enables statistical comparison of the variation in genetic diversity among a group of isolates (e.g., from a single country) with the variation in genetic diversity between groups of isolates (e.g., where isolates are derived from different countries).

**Figure 3:**
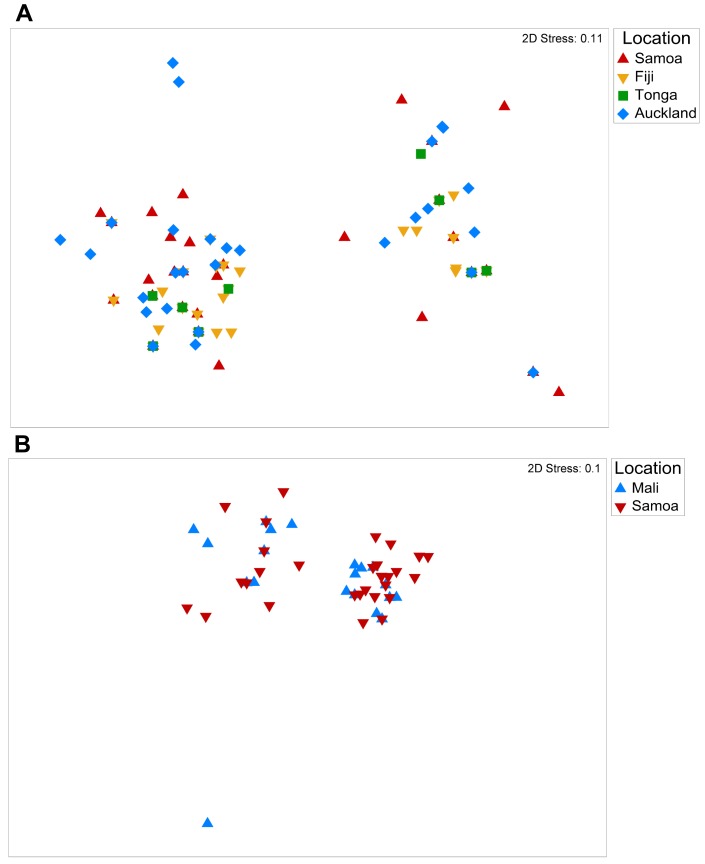
Multi-dimensional scaling map of *S. aureus* STs in (A) Southwest Pacific nations; and in (B) Samoa and Mali. Each map displays STs as points, color coded by country of isolation; the considerable overlap between STs isolated from different countries was confirmed by a lack of statistically significant difference in the variation of genetic diversity between groups of isolates from different countries.

Figure 3A shows the Southwest Pacific *S. aureus* population displayed as a two-dimensional map formed by multi-dimensional scaling of the genetic distance matrix [40]. CC75 strains were excluded from the map, because their marked divergence caused the remaining isolates to cluster; however, they were included in the multivariate analysis. The genetic diversity of *S. aureus* populations in each Southwest Pacific nation did not differ significantly from the genetic diversity of *S. aureus* populations in the other Southwest Pacific nations (PERMANOVA pairwise tests, *P*>0.05)(Table S1).

The lack of significant difference in the genetic diversity between *S. aureus* populations in neighboring Southwest Pacific nations could be due to mixture of *S. aureus* populations as a consequence of human travel within the region. We therefore compared the genetic diversity of the *S. aureus* sample from Samoa with the genetic diversity of a *S. aureus* sample from Mali [10], on the basis that human travel and migration between Samoa and Mali is likely a rare event. Interestingly, while 12 STs were unique to the Mali *S. aureus* sample and 21 STs were unique to the Samoa *S. aureus* sample (eight STs were shared), the genetic diversity within the *S. aureus* population from Samoa did not differ significantly from that within the *S. aureus* population from Mali (PERMANOVA, *P* = 0.42, Figure 3B).

### Global biogeography of *S. aureus*

**Figure 4:**
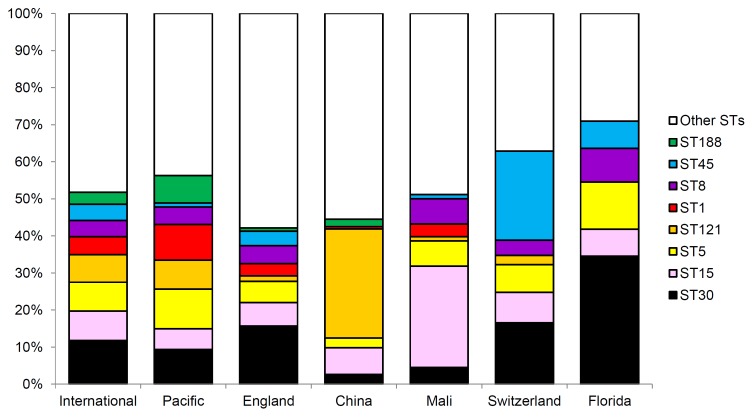
The frequency of isolation of the 8 predominant STs in the intercontinental dataset and for each location. Information regarding the frequency of isolation of North Carolina isolates was not available.

**Figure 5:**
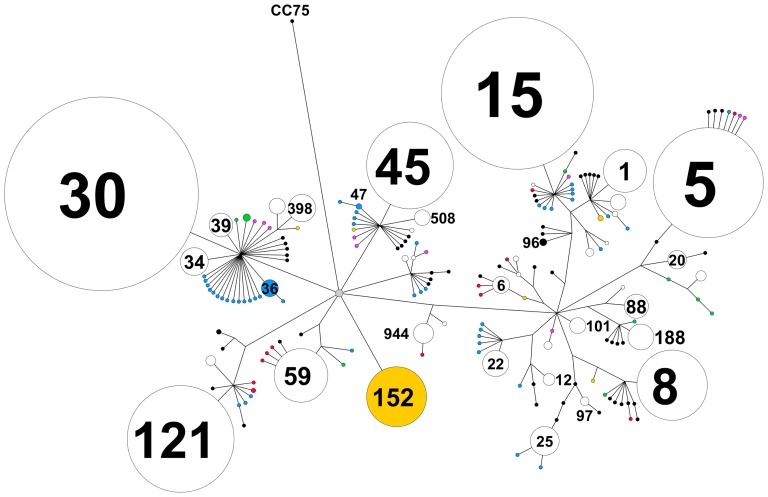
Phylogenetic network of intercontinental *S. aureus* isolates reconstructed from concatenated MLST gene nucleotide sequences using BEAST. Predominant STs are labeled and the size of each node is proportional to the number of isolates, adjusted for sample size; branch lengths have been modified for display purposes. STs found only in one location are colored: Southwest Pacific countries (black), STs found only in England (blue), STs found only in China (red), STs found only in Mali (yellow), STs found only in Florida (green) and ST found only in Switzerland (pink); STs found in more than one location are not colored.

Figure 4 shows that more than half of the isolates in the global dataset belong to one of eight predominant STs (ST30, ST15, ST5, ST121, ST1, ST8, ST45 and ST188). While ST152 strains have been frequently isolated only in Mali, other predominant STs are widely distributed. In England during the late 1990s, ST30 was predominant and ST121 was uncommon; in China during 2005, ST121 was predominant and ST8 was absent; and in Mali during 2005, ST15 and ST152 predominated and ST121 was uncommon. When ST diversity and abundance are viewed on a phylogenetic network (Figure 5) the range of unique STs becomes visible. Of interest is the fact that the majority of unique STs are in close proximity to the globally predominant STs indicating recent divergence from these abundant STs.

Given the lack of a significant difference between the Samoan and Mali populations, we performed a series of analyses to look for differences in genetic diversity between the Southwest Pacific population and populations previously reported from Asia, Europe, North America or Africa. Overall, there was a trend towards an association between country of origin and *S. aureus* genetic diversity (PERMANOVA, Pseudo F = 1.48, *P* = 0.06). Pairwise comparisons of *S. aureus* genetic diversity between countries demonstrated few statistically significant differences (Table S1). Statistically significant differences were only identified between *S. aureus* genetic diversity in England or Switzerland when compared with other countries.

In analyses of differences between continents, when *S. aureus* populations from England and Switzerland were grouped to represent Europe, the genetic variation of the European *S. aureus* population differed from the genetic variation of *S. aureus* from all other countries combined (PERMANOVA, *P*<0.01). Likewise, there was a significant difference in the genetic variation of *S. aureus* from Southwest Pacific countries in comparison with all other countries (PERMANOVA, *P*<0.01). These differences were likely due to a small number of STs in the *S. aureus* populations of all other countries that were not present in Europe or the Southwest Pacific, rather than the presence of divergent STs in those two regions. The STs absent from the Southwest Pacific *S. aureus* population are shaded in red in Figure S3. There were no significant differences between the genetic diversity of *S. aureus* in Asia, Africa or North America and other countries, respectively (PERMANOVA, Asia *P* = 0.16; Africa *P* = 0.37; North America *P* = 0.40).

**Figure 6:**
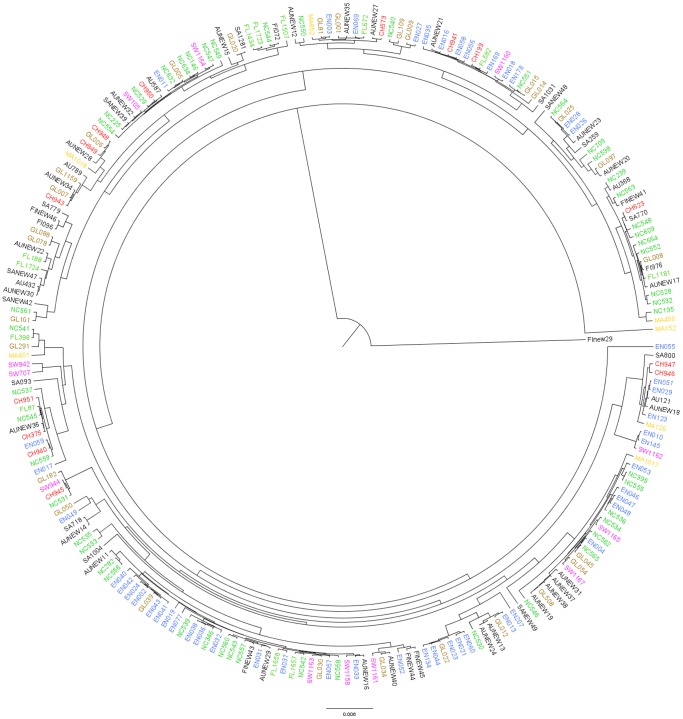
Intercontinental phylogeny of *S. aureus* reconstructed from concatenated MLST gene nucleotide sequences. The phylogeny is rooted on a CC75 strain isolated in Fiji; taxa are labeled with a prefix indicating their origin (AU, Auckland (black); CH, China (red); EN, England (blue); FI, Fiji (black); FL, Florida (green); GL, global, present on several continents (brown); MA, Mali (yellow); NC, North Carolina (green); SA, Samoa (black); SW, Switzerland (pink)) and a suffix indicating their ST number (e.g. EN040 is an ST40 isolate from England).

A phylogeny (Figure 6) constructed from STs from samples obtained from Auckland, China, England, Fiji, Florida, Mali, North Carolina, Samoa, and Switzerland reveals that the number of STs shared between at least two different continents (“globally dispersed STs”; brown labels on Figure 6) is small in comparison with the total number of taxa. However, as shown in Figure 5, most of the taxa found in only one location are closely related to one of the predominant, globally dispersed STs. Notable, when the tree is rooted on a divergent CC75 ST, is genetic homogeneity - with the exception of CC75 and ST152 - terminal branch lengths are short.

## Discussion

Knowledge of the genetic structure of *S. aureus* from the Southwest Pacific complements insights obtained from similar studies performed in different geographical regions including Asia, Africa, Europe, and North America. Key findings include the following: (i) predominant STs (and CCs) vary in distribution and frequency across small neighboring Southwest Pacific Island nations (Figure 1), and between countries on different continents (Figure 4); (ii) despite finding (i), the overall genetic diversity of *S. aureus* is similar between different countries and across wide geographical divides (Figure 3B); (iii) novel STs reach frequencies sufficient to allow their detection even when sample size, relative to the human population size, are limited (Figure 5); (iv) phylogenies marked by short terminal branch lengths demonstrate that extant STs have diverged recently from predominant and globally distributed STs (Figures 5 and 6); and (v) the markedly divergent CC75 *S. aureus* strains are present in multiple locations (Figure 6).

The most striking example of differences in ST distribution between countries was the absence of ST1 in Fiji, given that ST1 was common in Auckland and Samoa. However, the genetic diversity of *S. aureus* within Fiji was similar to that found in other Southwest Pacific nations and more distant geographical regions. Furthermore, Fiji also harbors STs that belong to the same clonal complex as ST1. Many examples of differences in the distribution in STs have been reported (e.g., the high prevalence of ST152 in Mali [10]) and comparison of the STs present in widely distant countries (Figure 4) suggests that *S. aureus* populations differ between continents (e.g., CC121 predominates in Asia [8], [9], whilst CC30 is common in Europe [6], [27]).

Maynard Smith, et al [43], showed the importance of examining the extent of genetic diversity (as well as abundance) when describing bacterial population structure: in the current study we performed statistical comparisons of the genetic diversity of *S. aureus* populations described in different countries. Despite unevenness in the distribution of STs among Southwest Pacific Island nations there was no significant difference between the genetic diversity of *S. aureus* populations from different Southwest Pacific Island nations. Furthermore, the genetic diversity of *S. aureus* populations in Southwest Pacific Island nations did not differ significantly from the genetic diversity of *S. aureus* populations from such distant locations as Mali and North America. Overall, the uniformity in the level of genetic diversity across different parts of the globe is a consistent and notable finding arising from multiple studies.

One of the major factors giving rise to differences in ST distribution is the capacity for novel STs to arise and reach a frequency sufficient for identification in studies of small sample sizes (relative to the size of human populations). The global phylogeny of *S. aureus* (Figure 6) shows that novel STs overwhelm STs that are globally distributed, and that novel STs are not biased toward any one lineage. The most prevalent ST in a given geographical region was often the focal type from which new types (often single locus variants) were derived. For example, for CC5 in the Southwest Pacific, where ST5 was the most prevalent ST, novel CC5 variants were more common than in other regions. Similarly, in England CC30 was the most prevalent and diverse lineage; in Asia, CC121 was more diverse than in England, or Mali (where CC121 was found at lower frequency with limited diversity).

Also notable from analysis of the global dataset are the short terminal branches that define the tips of the phylogenetic tree (Figure 6). Leaving aside the rare, but divergent, CC75 strains, extant *S. aureus* isolates appear to have diverged recently from a common ancestor. This raises the possibility that CC75 is a relic of an ancestral *S. aureus* population that is evolving into a new species: *S. argenteus* [17]. It is surprising that further divergent lineages have not been discovered.

In contrast to our findings, a phylogeny derived from genomic sequences from CC121 strains collected from around the globe [44] showed marked geographical structure. This shows that CC121 STs have not simply spread from region to region as a result of recent human travel, which would remove geographical structure; rather, CC121 *S. aureus* strains appear to have evolved in sufficient isolation to give rise to geographical structure between different continents. Analyses of the other globally distributed CCs, using methods that examine genomes with greater resolution than that provided by MLST, will improve understanding of this globally important pathogen.

Overall, our comparisons demonstrate that statistically significant genetic variation between *S. aureus* populations derived from different countries is occasionally encountered. However, these differences are not substantial and are likely to represent variation among novel variants that have arisen from predominant STs and increased in frequency to enable detection. The relative genetic homogeneity of *S. aureus* as a whole suggests that predominant CCs are members of a lineage that has recently come to prominence.

## Supporting Information

Figure S1 **Rarefaction curves of** ***S. aureus*** **samples from Auckland (n = 244), Fiji (n = 109), Samoa (n = 96) and Tonga (n = 18).** (Figure S1A) The number of isolates required to identify unique STs; (Figure S1B) The number of isolates required to identify the number of operational taxonomic units (OTU - groups of related *S. aureus* STs that vary by less than 16/3198, 0.005 nucleotides – similar to the variation seen within most clonal complexes) in the population. The number of OTUs in each sample was saturated - the number of unique STs in each sample did not increase with increasing sample size. Rarefaction analysis was performed using MOTHUR v1.25.1 [46].
(TIF)

Figure S2 **Phylogeny of** ***S. aureus*** **isolates from the Southwest Pacific rooted on CC75** ***S. aureus*** **isolates.** The phylogeny was reconstructed using BEAST v1.7.2 [37]. Novel isolates, dispersed throughout the phylogeny, are shaded grey. AU is Auckland, FI is Fiji, SA is Samoa, TO is Tonga; numbers refer to ST designation.
(TIF)

Figure S3 **2D-multidimensional scaling map of** ***S. aureus*** **genetic variation amongst isolates from Southwest Pacific nations (Auckland, Fiji, Samoa, Tonga) in comparison with isolates from other countries (China, England, Mali, Switzerland, USA; PERMANOVA,** ***P*** **<0.01).** The statistically significant differences in genetic variation between groups of *S. aureus* isolates, is potentially related to STs in the shaded region, which were not identified in the current study.
(TIF)

Table S1 **Results of pairwise statistical testing comparing the genetic variation of** ***S. aureus*** **populations between different countries.** The genetic distances between all non-duplicate STs from each country (described in the current study and by Feil, et al [6]; Fan, et al [8]; Ruimy, et al [10]; Sakwinska, et al [27]; Fowler, et al [12]; and Lamers, et al [28]) were incorporated into a distance matrix and hypothesis tests were performed using PERMANOVA. No statistically significant comparisons between the genetic variation in an individual country and all of the remaining countries were detected. Each statistical test is independent and correction for multiple analyses has not been applied [41], [45].
(DOCX)

Data S1 **Non-duplicated concatenated MLST nucleotide sequences of intercontinental STs and allele designations of isolates obtained from the Southwest Pacific.**
(ZIP)
