# Supplementary figures and images for: The Genetic Structure of Staphylococcus aureus Populations from the Southwest Pacific
Source: PLoS One. 2014 Jul 8;9(7):e100300. doi: 10.1371/journal.pone.0100300 (PMC4086726; doi:10.1371/journal.pone.0100300)

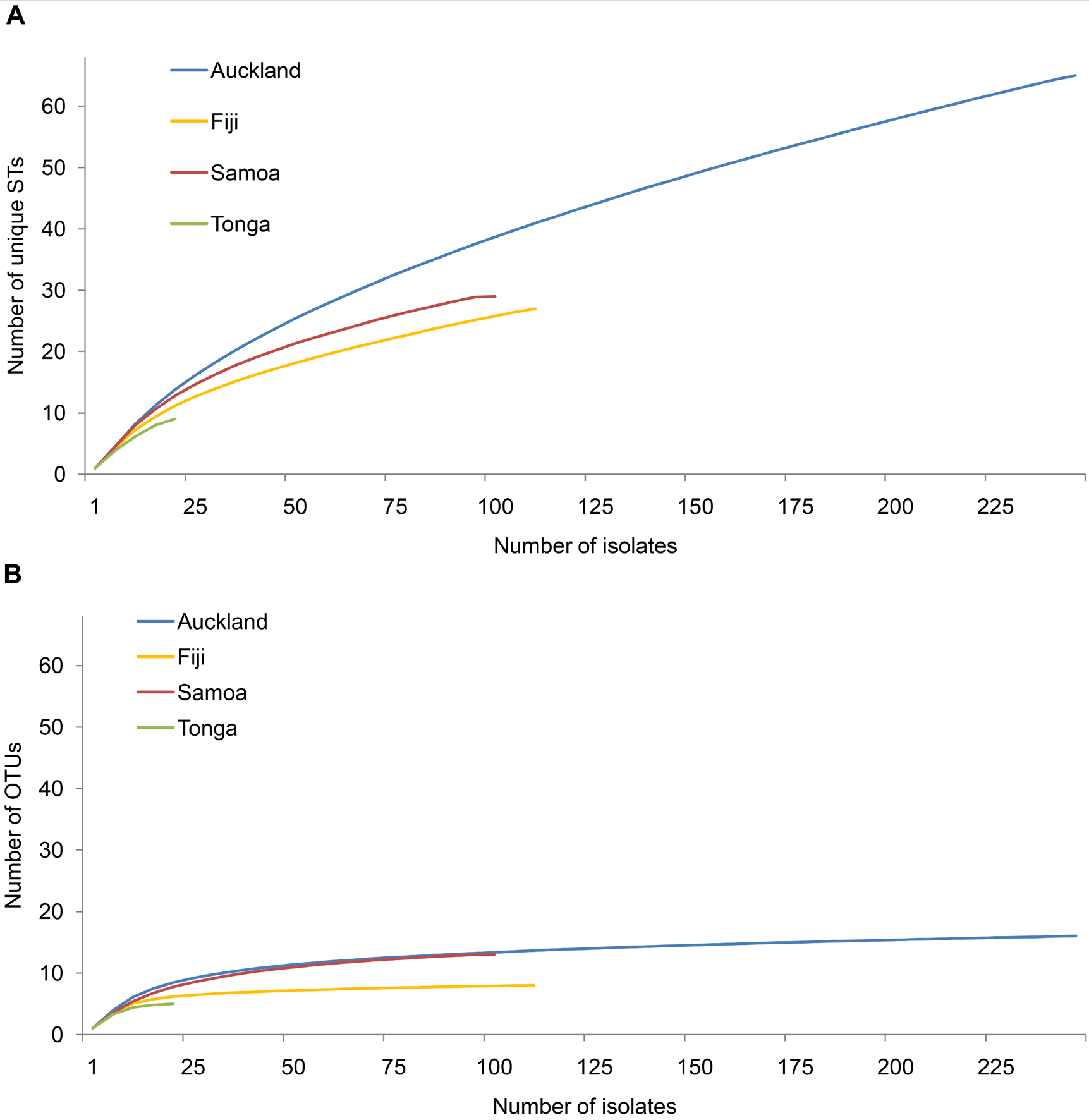

Supplement: Figure S1 — Rarefaction curves of S. aureus samples from Auckland (n = 244), Fiji (n = 109), Samoa (n = 96) and Tonga (n = 18). (Figure S1A) The number of isolates required to identify unique STs; (Figure S1B) The number of isolates required to identify the number of operational taxonomic units (OTU - groups of related S. aureus STs that vary by less than 16/3198, 0.005 nucleotides – similar to the variation seen within most clonal complexes) in the population. The number of OTUs in each sample was saturated - the number of unique STs in each sample did not increase with increasing sample size. Rarefaction analysis was performed using MOTHUR v1.25.1 [46]. (TIF) [file pone.0100300.s001.tif]

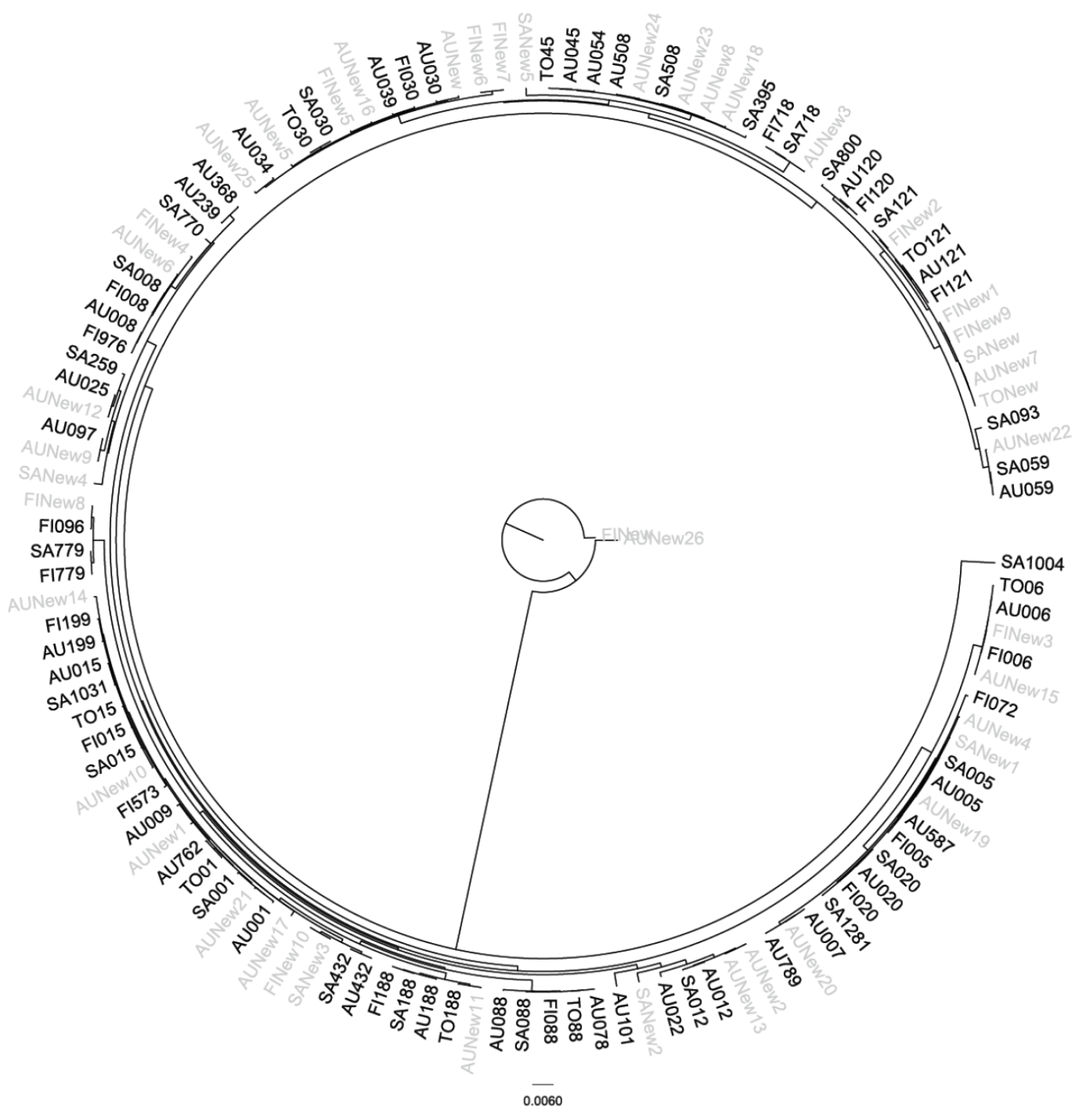

Supplement: Figure S2 — Phylogeny of S. aureus isolates from the Southwest Pacific rooted on CC75 S. aureus isolates. The phylogeny was reconstructed using BEAST v1.7.2 [37]. Novel isolates, dispersed throughout the phylogeny, are shaded grey. AU is Auckland, FI is Fiji, SA is Samoa, TO is Tonga; numbers refer to ST designation. (TIF) [file pone.0100300.s002.tif]

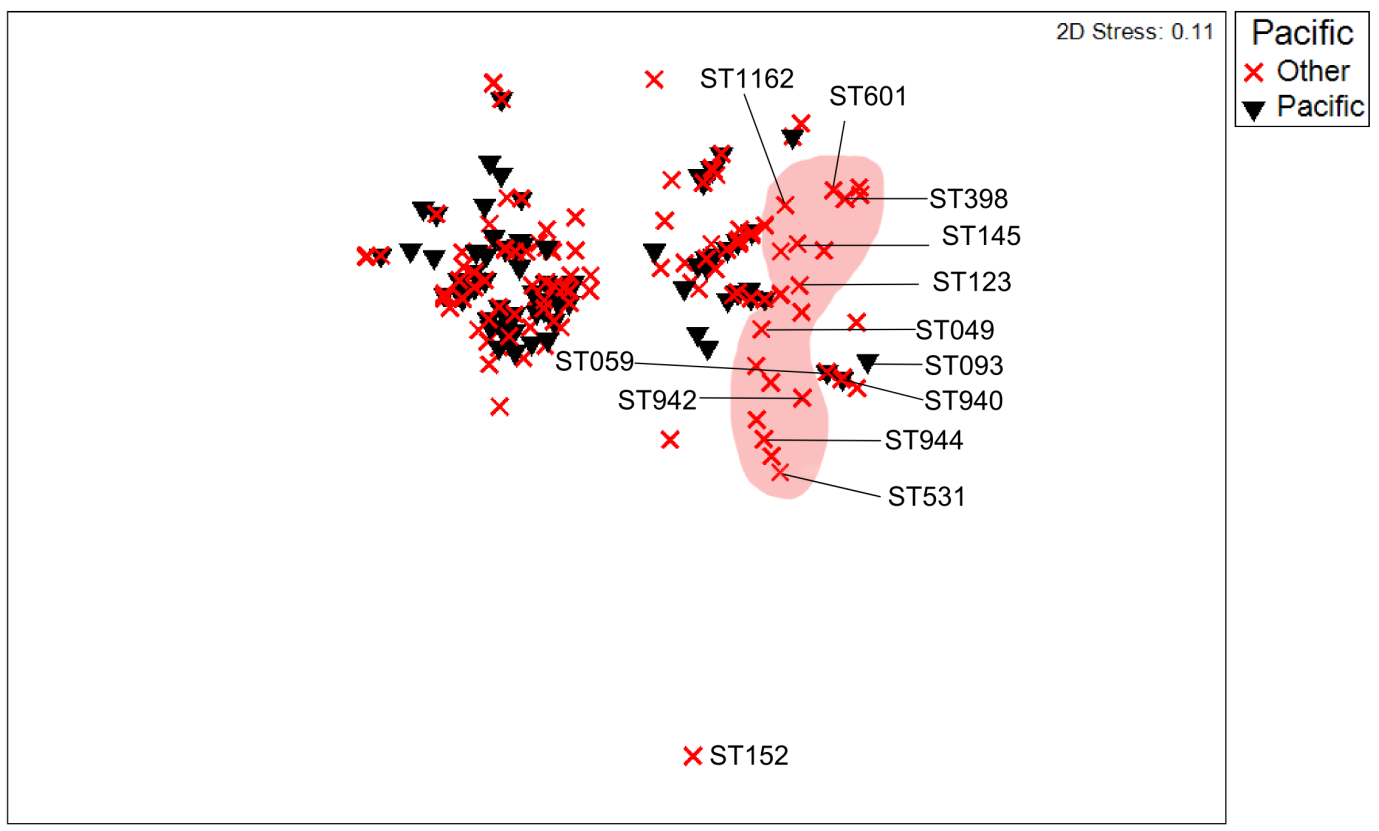

Supplement: Figure S3 — 2D-multidimensional scaling map of S. aureus genetic variation amongst isolates from Southwest Pacific nations (Auckland, Fiji, Samoa, Tonga) in comparison with isolates from other countries (China, England, Mali, Switzerland, USA; PERMANOVA, P <0.01). The statistically significant differences in genetic variation between groups of S. aureus isolates, is potentially related to STs in the shaded region, which were not identified in the current study. (TIF) [file pone.0100300.s003.tif]
