# Supplementary material for: The Genetic Structure of Staphylococcus aureus Populations from the Southwest Pacific
Source: PLoS One. 2014 Jul 8;9(7):e100300. doi: 10.1371/journal.pone.0100300 (PMC4086726; doi:10.1371/journal.pone.0100300)
Supplement: Table S1 — Results of pairwise statistical testing comparing the genetic variation of S. aureus populations between different countries. The genetic distances between all non-duplicate STs from each country (described in the current study and by Feil, et al [6]; Fan, et al [8]; Ruimy, et al [10]; Sakwinska, et al [27]; Fowler, et al [12]; and Lamers, et al [28]) were incorporated into a distance matrix and hypothesis tests were performed using PERMANOVA. No statistically significant comparisons between the genetic variation in an individual country and all of the remaining countries were detected. Each statistical test is independent and correction for multiple analyses has not been applied [41], [45]. (DOCX) [file pone.0100300.s004.docx]

| Country | AU | FI | SA | TO | CH | EN | MA | SW | NC |
| --- | --- | --- | --- | --- | --- | --- | --- | --- | --- |
| Auckland (AU) |  |  |  |  |  |  |  |  |  |
| Fiji (FI) | 0.38 |  |  |  |  |  |  |  |  |
| Samoa (SA) | 0.93 | 0.63 |  |  |  |  |  |  |  |
| Tonga (TO) | 0.73 | 0.93 | 0.88 |  |  |  |  |  |  |
| China (CH) | 0.11 | 0.56 | 0.40 | 0.78 |  |  |  |  |  |
| England (EN) | 0.04 | 0.08 | 0.07 | 0.65 | 0.04 |  |  |  |  |
| Mali (MA) | 0.23 | 0.30 | 0.42 | 0.90 | 0.31 | 0.16 |  |  |  |
| Switzerland (SW) | 0.01 | 0.04 | 0.05 | 0.44 | 0.13 | 0.22 | 0.24 |  |  |
| USA – Nth Carolina (NC) | 0.14 | 0.10 | 0.19 | 0.64 | 0.23 | 0.21 | 0.42 | 0.27 |  |
| USA – Florida (FL) | 0.71 | 0.46 | 0.79 | 0.80 | 0.81 | 0.22 | 0.68 | 0.17 | 0.87 |
